# Do Strategies to Improve Quality of Maternal and Child Health Care in Lower and Middle Income Countries Lead to Improved Outcomes? A Review of the Evidence

**DOI:** 10.1371/journal.pone.0083070

**Published:** 2013-12-09

**Authors:** Zoe Dettrick, Sonja Firth, Eliana Jimenez Soto

**Affiliations:** School of Population Health, University of Queensland, Brisbane, Queensland, Australia; University of Cape Town, South Africa

## Abstract

**Objectives:**

Efforts to scale-up maternal and child health services in lower and middle income countries will fail if services delivered are not of good quality. Although there is evidence of strategies to increase the quality of health services, less is known about the way these strategies affect health system goals and outcomes. We conducted a systematic review of the literature to examine this relationship.

**Methods:**

We undertook a search of MEDLINE, SCOPUS and CINAHL databases, limiting the results to studies including strategies specifically aimed at improving quality that also reported a measure of quality and at least one indicator related to health system outcomes. Variation in study methodologies prevented further quantitative analysis; instead we present a narrative review of the evidence.

**Findings:**

Methodologically, the quality of evidence was poor, and dominated by studies of individual facilities. Studies relied heavily on service utilisation as a measure of strategy success, which did not always correspond to improved quality. The majority of studies targeted the competency of staff and adequacy of facilities. No strategies addressed distribution systems, public-private partnership or equity. Key themes identified were the conflict between perceptions of patients and clinical measures of quality and the need for holistic approaches to health system interventions.

**Conclusion:**

Existing evidence linking quality improvement strategies to improved MNCH outcomes is extremely limited. Future research would benefit from the inclusion of more appropriate indicators and additional focus on non-facility determinants of health service quality such as health policy, supply distribution, community acceptability and equity of care.

## Introduction

There is a strong evidence base of interventions that are effective in preventing maternal, neonatal and child mortality in lower and middle income countries (LMICs)[1-3]. However underlying the estimates of effectiveness is the assumption that the interventions delivered are of good quality. While many countries have been successful in increasing utilisation of services through demand promotion programs, these efforts can be accompanied by poor or declining quality of service[4].

A number of studies have examined tools and strategies to improve the quality of maternal, newborn and child health (MCNH) services in developing countries[5,6] and the impact of their implementation on quality of care [7-9]. They have found that while there is some evidence that quality improvement strategies can lead to improvements in process indicators, such as clinical knowledge and practice, these analyses do not demonstrate that the observed improvements impact on wider health outcomes[5,6]. 

Other studies have assessed the extent to which packages of MNCH interventions, including quality improvement activities, reduce mortality [10,11]. However as there was no attempt to define, measure and assess changes in quality of care, a causative relationship could not be established. Recent reviews of clinical audits [7,12,13] and training in emergency obstetric care (EMOC) [9] note that while it is assumed that these strategies will lead to better outcomes, evidence of impact due to improved quality is lacking. 

**Figure 1:**
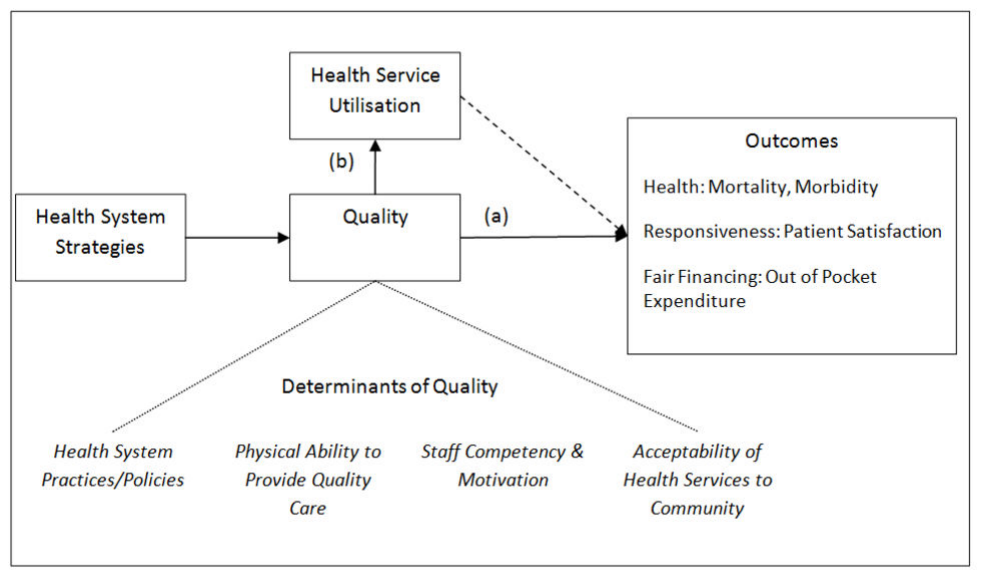
Conceptual Framework of Pathways between Strategies, Quality and Outcomes. Solid lines represent presumed direct relationships while indirect relationships are represented by dashed lines. Broad determinants of quality that may be targeted by strategies are shown at the bottom of the figure. Health system strategies lead to improved quality which can either (a) directly lead to improved outcomes or (b) lead to improved health service utilisation which may indirectly lead to improvements in outcomes. An example of pathway (a) may be a maternal death audit, targeting the competency of staff, resulting in higher use of correct management techniques leading to lower mortality. An example of pathway (b) might be renovation of primary care facilities leading to higher community acceptability, resulting in more children being brought for treatment and thus a reduction in deaths due to treatable causes.

We attempted to address this evidence gap by conducting a systematic review of the literature, focusing on evidence quantitatively linking strategies to improve quality of MNCH in LMICs to both quality of care and health system outcomes. To facilitate the discussion, Figure 1 illustrates such pathways, as well as the broad determinants of quality of care, drawing on pre-existing theoretical frameworks [14-17]. 

## Methods

As with other complex health interventions [18], this topic poses several challenges to the definition of inclusion and exclusion criteria. While there are established guidelines regarding the actions that comprise quality clinical care for particular MNCH interventions[19], measuring the quality of other elements of the health system essential to health service delivery (e.g. infrastructure or staff motivation), is more difficult[6,16]. MNCH services are highly diverse, and as a result there can be significant variation in the type of health system strategies employed, and the measures used to determine a successful outcome in different settings. 

These factors make it difficult to standardize definitions and indicators for inclusion in the review, as the framework used to examine quality may vary by study. No pre-existing protocols were available on which to draw. We started with a broad search strategy that aimed to identify studies that used different definitions and measures of quality without imposing subjective filters as to what constituted “quality”. Search terms related to MNCH, “quality” and LMICs (full terms listed in Appendix – Table S1) were used to search MEDLINE, SCOPUS and CINAHL databases on February 3, 2012. No language filters were applied, however publication years were limited to post 1990. 

**Figure 2:**
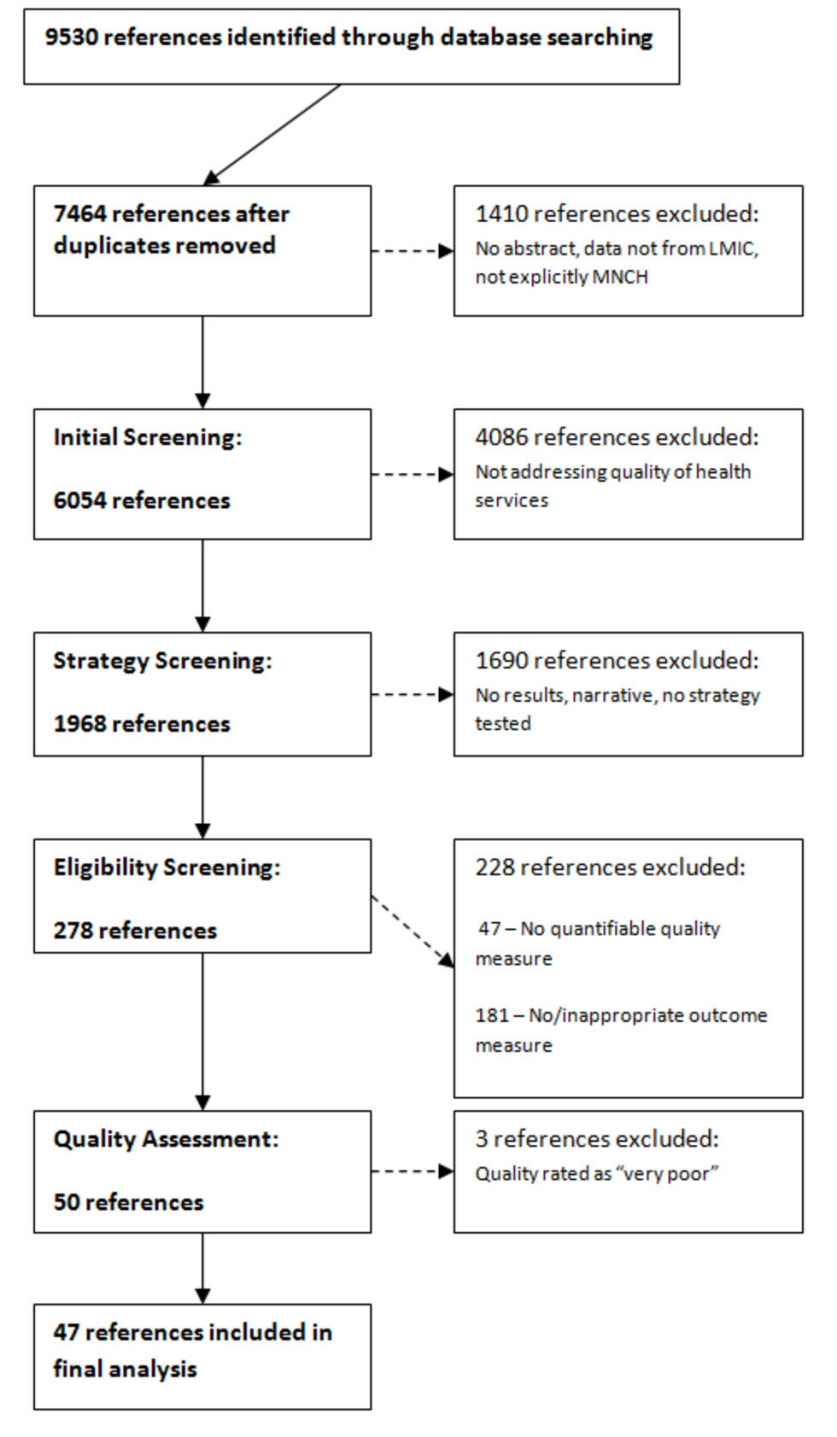
Study selection process.

After removing duplicates, this initial search provided a total of 7464 references (see Figure 2). We then removed any references not specifically evaluating MNCH, those that were based on data from high income countries or that lacked an abstract. The remaining references were screened to examine the extent to which the study discussed the delivery of MNCH services. Articles that did not examine any aspect of health service delivery, such as those reporting drug trials were excluded. This process resulted in 1968 references. Since we wished to examine the impact of health system strategies on quality, we then excluded a further 1690 references that were narratives, did not explicitly state improved quality of care or improved service delivery as a goal of the study, failed to test suggested strategies or did not report results.

Two reviewers independently assessed the 278 remaining references. They were screened for the presence of at least one measure of quality and one measure of health system outcomes. This was necessary to demonstrate that change had or had not occurred following strategy implementation. To avoid potential bias, as a result of our subjective views on appropriate measures of health service quality, we accepted any measure stated by the authors as representing improved quality, as long as it was quantifiable. Definitions of health system outcomes were based on the framework set out in the World Health Report 2000 [20]. Studies were only included if they reported quantifiable measures of health (e.g. mortality or morbidity), fair financial contribution (e.g. out of pocket expenditure) or responsiveness (e.g. patient satisfaction). Based on initial screening studies for which authors suggested indirect improvements in health outcomes, via quality improvement leading to increased utilisation of health services (see Figure 1), were also included. Disagreements in classification between reviewers were resolved through discussion. This led to 50 references being examined by the reviewers. No additional references were identified from bibliographic sources.

 The methodological quality of the remaining studies was assessed using a checklist of criteria based on the PRISMA, CONSORT and STROBE statements. Checklist items differed depending on the experimental design used. If there were no major issues based upon the checklist, criteria were assigned a value of 2. Where the result of the checklist indicated some issues it was assigned a value of 1. If the majority of indicators were not present, were poorly demonstrated or otherwise inappropriate, criteria were assigned a value of 0. A simple algorithm was then used to create a weighted total out of 10, with double weight applied to selection, methodology and results criteria.

Studies receiving a score of 9-10 were classified as being of “good” quality, 8-9 as “moderate” quality and 7-8 as “poor” quality. Studies receiving a score below 7 were excluded from the review. As a result, 47 references were included in the final review. 

For each study included in the final analysis, data was extracted with regard to study type, sample size, setting and context. Also extracted were the details of the strategy being implemented (e.g. training in ANC, maternal death audit), the quality and outcome measures used, any quantitative results, and the author’s conclusions. The small number of studies, the diversity of experimental designs and strategies used precluded a full meta-analysis and quantitative evaluation. Instead, we present a narrative review of the key themes arising from the literature. Extracted data were used to group studies according to similarities in structure and outcomes. For each nominated outcome it was recorded if the study reported positive, negative, neutral or mixed effects in order to judge a strategy’s success. Detailed examination of the full texts was then used to identify narrative elements related to the implementation or consequences of the strategy. An ethics statement was not required for this work. 

## Results

47 references were included in the analysis, representing 45 unique studies. A summary table of included studies is available in Appendix – Table S2. Most studies were from African contexts, with Latin America and South Asia being the other frequent areas of study. Overwhelmingly, interventions were initiated by non-government organisations and undertaken within the public health system. Most interventions precluded the use of randomisation and blinding, and sample sizes were usually limited to a small number of districts or specific facilities. As a result the level of evidence was limited. The majority of studies (34) consisted of observational pre/post designs without control groups, 4 were observational studies with control groups and 7 were randomised control trials (RCT). While all but one of the RCTs were rated as being of “good” quality, most of the observational studies were only of “moderate” quality. The most frequent issue noted was the lack of reporting of participant characteristics to control for selection bias in pre/post designs. 

The measures of quality and outcomes used in the studies varied considerably. Quality was often measured as adherence to appropriate clinical practices or provision of health services (availability of drugs, use of specialised equipment). Case fatality rates were frequently used as a measure of quality in studies with a strong institutional focus, such as EMOC. Other measures of quality included training scores, avoidable mortality and client satisfaction. All but one study[21] reported improvements in at least one of the quality measures used. 

**Figure 3:**
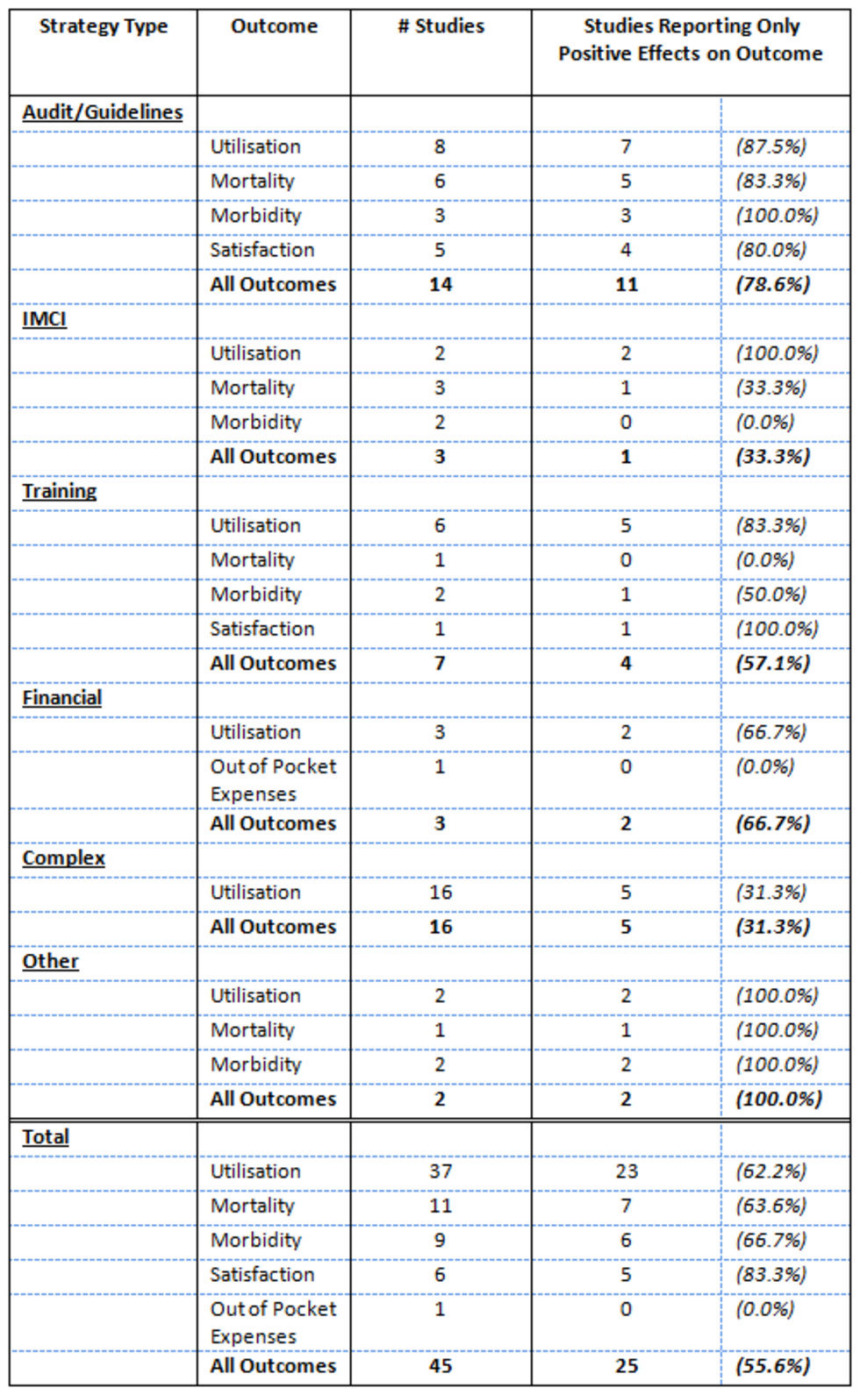
Proportion of Studies Reporting Positive Effects for Given Outcomes by Strategy Type. Notes: Effects were considered positive if the authors reported the result as being beneficial. If multiple indicators existed for the same type of outcome it was only considered positive if all results were reported as being beneficial.

The most common outcome reported was utilisation of services, with 23 studies including this as their only outcome measure and another 14 including it as one of multiple outcomes. Of direct measures of health system outcomes, mortality was a frequent indicator as was the prevalence or incidence of specified morbidities. Other indicators used were client satisfaction and out-of-pocket expenditure on health. Figure 3 tables the reporting of positive effects for outcomes by strategy type.

 Of the 45 studies, only 25 reported positive effects on all outcomes measured; however 41 reported positive effects on at least one outcome. No outcome was uniformly positive in all studies. Outcomes were more likely to show positive effects if they were directly related to the quality measure under improvement. Strategies to improve client satisfaction were more likely to report positive outcomes on utilisation, and strategies involving changes in clinical practices were more likely to report positive outcomes on mortality. The type of strategies utilised by the studies fell into six broad groups:

### Audits, Feedback and Guidelines

These studies used methods incorporating elements of an evaluation-action cycle to identify areas for improvement and develop strategies to overcome problems. The exact methods used were diverse, including the use of clinical audits[22-25], guidelines [26,27], public feedback through ‘scorecards’ [28,29] or a combination of these methods with the addition of specialised task groups and training [30-35]. In all fourteen studies quality measures improved overall, although the results were not uniform. All of the studies reported positive effects on at least one outcome measure, however three demonstrated a mismatch in the relative success of r mortality and utilisation outcomes, suggesting other determinants may be more important to one set of outcomes. The strongest results were associated with participatory methodologies involving both staff and administration and targeting of specific items for improvement. 

### IMCI

The Integrated Management of Childhood Illness (IMCI) is a UNICEF/WHO developed initiative aimed at improving the management of priority childhood illnesses such as diarrhoea, malaria and pneumonia. A key element of this approach involves the introduction of treatment guidelines, adherence to which is then used as a measure of clinical quality. Three IMCI related studies were included, one regarding its implementation in Bangladesh [36,37], another in Benin [38] and a third examining the overall impact of the Accelerated Child Survival and Development (ACSD) program (which included IMCI, ANC and Immunisation programs) in West Africa[39]. 

Adherence to guidelines and facility utilisation improved in the two studies examining local implementation, however mortality was reported to have decreased only in Benin. The assessment of IMCI programs included in the ACSD reported no impact on either mortality or morbidity outcomes. However it also found little increase in the utilisation of IMCI services, and suggested that this may have limited the effect of improved quality. 

### Training

Training of health staff is a commonly implemented strategy to improve the quality of health services, however the bulk of studies identified in the initial search measured only process indicators, such as knowledge pre and post training. Seven studies of the effect of training strategies were included in the final analysis. The subject matter included in the training stratigies included pregnancy care [40,41], delivery care [42,43], immunisation [44,45] and client rights and communication [46]. Most studies demonstrated improvements in quality, but measures of health system outcomes were less consistent. Two of these studies [40,45] are heavily limited by their use of a pre-post test scores as a measure of quality change – previous studies have noted that while they indicate a change in provider knowledge, sustained changes in provider practice are not assured [8]. 

### Financial

These strategies involved changes to financial mechanisms to provide funding for quality improvement efforts. One study [21] examined the introduction of community based insurance schemes to provide necessary funds to improve service quality. Other studies [47,48] reported on the use of monetary incentives to improve specific aspects of care. While general utilisation of facilities tended to increase, quality and use of specialised services were more variable. One study [21] recorded declines in clinical quality and another [48] reported significantly lower levels of institutional delivery than in control areas. The only study reporting out of pocket expenditure found a higher per capita expenditure overall, but lower expenditure in the poorest quintile. There were no studies examining the impact of these types of financial strategies on quality outside West and Central Africa.

### Complex

Studies that called for the implementation of a number of strategies as part of a larger program were classed as ‘complex’. Most included substantial facility and equipment upgrades to improve the quality of services on offer. Other common elements included training, quality monitoring, referral systems and community mobilisation. 

The majority of studies in this group focused on improving EMOC services. Two major initiatives dominate the studies in this category ; the PMM (Preventing Maternal Mortality) projects [49-53] focused on individual referral hospitals in Africa during the 1990s while the AMDD (Averting Maternal Death and Disability) projects [54-60] took a district based approach in a diverse range of sites a decade later. Of the two other studies examining EMOC, one examined a similar hospital rejuvenation project in Ethiopia [61] while the other examined the impact of a national level program to improve EMOC facilities in Peru[62]. Quality measures in these studies generally improved, although the magnitude of improvement was highly heterogeneous. No clear pattern was seen overall in terms of utilisation (the sole outcome measure used in these studies). 

 The remaining studies examined the results of minor changes to the management, training and monitoring of primary health care in a rural Indian district[63], and evaluated the radical restructure of the primary health care model in Guatemala[64]. Both studies examined the role of systemic rather than facility based changes, and demonstrated positive outcomes for both quality and utilisation.

### Patient-Provider Interaction

Two other studies examined strategies involving how patients interacted with providers. The first involved the provision of personalised maternal care and follow up by assigning patients to one provider for the duration of their care [65], while the second examined the use of group ANC sessions rather than individual care[66,67]. Both studies reported improvements in quality, and in outcome measures such as utilisation and prevalence of low birth weight infants.

The large majority of strategies aimed to improve staff competency and motivation. There was considerable overlap with strategies addressing the physical ability to provide services with many initiatives aiming to improve the quality of facilities and of staffing at the same time. While these studies generally reported improved mortality indicators, outcomes related to service usage were mixed. There were comparatively few strategies to improve health system policies and acceptability of health services, and these were usually implemented in conjunction with other elements. No strategies targeted all broad determinants, and the majority targeted only one or two. As a result, while there is a certain level of evidence regarding quality improvement strategies aimed at staff and facilities, information is limited about the potential benefit of strategies incorporating changes to health policies or efforts aimed at addressing specific community needs.

Although multiple studies examine the impact of increasing physical inputs such as equipment and drugs on the quality of services, no strategies to improve underlying issues with logistics or distribution were identified. Similarly, notwithstanding the large market share of private providers in developing countries [68,69] few studies examined strategies to improve quality within the private sector. No studies were identified that examined the use of accreditation, administrative regulation and oversight or public/private partnerships. Additionally, only one study was identified that explicitly examined the impact of improved quality on equity in healthcare [48].This is a concern given the concentration of ill health among disadvantaged population groups.

While the diversity of the included studies was large, several key themes emerged regarding the implementation needs and expected effect of quality improvement strategies. 

### Importance of perceived quality to increased utilisation of services

 Despite the differences in approach, studies that focused on patient perceptions tended to lead to increased service use. Jafari et al [66,67] found that participants in the group ANC sessions were more satisfied, attended more ANC sessions and were more likely to refer new women to the facility, than those with usual care. Somewhat paradoxically, Marin and colleagues [65] found that a change in policy to assign each patient to a named provider responsible for both pregnancy and delivery care increased both utilisation and continuity of care. This suggests that the number of staff available may be a weaker determinant of perceived quality compared to the way in which these staff interact with patients. Similarly, a study aimed at changing the way in which patients were perceived by hospital staff [46] found marked improvements in patient satisfaction with delivery care. It also led to an increase in the total number of complicated deliveries, as the change in attitude made traditional birth attendants more likely to offer, and patients more willing to accept, hospital referrals. 

Conversely, poor perception of facilities or staff competence could decrease utilisation as was seen in an AMDD facility in Vietnam [57] following the report of a maternal death. Perceived incompetence was similarly an issue in the rural Indian district examined by Barua et al [63] where implementation of strategies to improve equipment and staff supervision saw a three to fourfold rise in attendance at outreach sessions. During the implementation of an IMCI program in Bangladesh [37] utilisation and quality of care at IMCI primary facilities improved, but distrust of referral facilities meant few severe cases received appropriate treatment. Subsequent modification of IMCI guidelines to increase the level of treatment available at the primary facilities lead to a fivefold increase in the number of children seeking treatment for severe pneumonia[27]. 

### Potential for conflict between patient satisfaction and clinical quality

However the expectations of clients did not always align with clinical best practice. Contrary to expectations, the inclusion of lay support during delivery in the guidelines trialled by Aghlmand et al [26] was rejected by patients in favour of trained personnel only. Similarly, despite improving clinical quality, a quality auditing strategy in Ecuador [32] failed to increase client satisfaction and utilisation.

The reverse is also true – client satisfaction and facility use could increase even if quality as measured by clinical criteria did not. Ouma et al [41] found a significantly greater proportion of women in the intervention areas rated the quality of ANC services as very satisfactory, despite the worsening in the measures of quality care following staff training. In the trial of obstetric risk insurance in Mauritania[21] utilisation of intervention facilities increased substantially despite declines in the clinical quality of both ANC and delivery care. 

### Impact of quality improvements can be lost or masked when financial access is poor

The potential of quality improvement strategies to improve outcomes was limited in financially vulnerable populations. The use of audits to improve obstetric referrals in Angola [24] caused impressive reductions in case fatality, but most women continued to opt for risky home deliveries rather than incur substantial debts to pay for advanced hospital treatment. Similar patterns were seen in Malawi following the introduction of maternal death audits[22], and despite major upgrades to equipment, facility and staff the number of complicated deliveries in Nigerian PMM projects [49,51-53] declined in all sites.

Balancing the willingness of the community to pay for services and the funding needs of the facility to maintain quality can be difficult. The trial of a new financial system at one facility in the Democratic Republic Of Congo [48] was associated with increases in structural and user perceived quality. However a quarter of the increased revenue at the facility came from increased user fees – raising potential equity issues. In contrast the high per capita costs of the restructured health system reported by Fort et al [64] made extension of the program difficult to justify despite impressive improvements in quality and mortality.

### Financial incentives do not always lead to improved quality

Providing additional funds to staff was not sufficient to improve the quality of their performance. Renaudin et al [21] noted that despite the provision of substantial financial performance incentives for staff, clinical quality declined. Further analysis found that the positive effect of the incentive was offset by increased workload and reductions in opportunities for ‘under the table’ payments and private sector work. 

In the ‘P4P’ program in Rwanda [47] provider payments were linked to specific health outcome indicators. To ensure that quality would be maintained, the amount each provider received was adjusted by a measure of clinical quality. As a result outcomes with high per-case incentives, such as institutional delivery, increased, as did those that helped contribute to the score of clinical quality, such as ANC care. For less remunerative indicators such as immunisation, coverage remained stagnant or even fell. 

### Need for high level support

Efforts to improve quality must be accompanied by coordinated support from policy and administrative levels if systemic issues preventing progress are to be overcome. Youngleson et al [35] attribute much of their success to the ability of administration to implement changes in policies and resource allocation within a short time frame. The AMDD project in Nepal [59] noted that the national policy allowing delegation of key obstetric first aid procedures to nurses was essential to the program impact. Two other studies [34,63] emphasise the critical role that formal support from state and district health officers played in ensuring long term commitment and support for the quality improvement initiatives.

During the evaluation period of the *Proyecto* 2000 program in Peru [62] the chaos resulting from the decentralisation and partial re-centralisation of public health services in hampered strategy implementation. Failure to secure timely provision of drugs and equipment [30,60], frequent reassignment of staff [31,56,58,61] and delays in implementing critical policy changes [37,39] are other issues reported stemming from poor policy decisions at higher levels.

### The potential for local level initiatives

In the absence of high level support local commitment can improve outcomes. The majority of audit and feedback based strategies did not involve large monetary investments, but instead enabled facilities to make the most of their existing resources [22,23,25-27,29-34]. Leigh et al [50] note that locally suggested equipment substitutions reduced costs and allowed for quicker implementation of new procedures due to easier procurement processes. Rescheduling of outreach sessions improved multiple issues surrounding access, supervision and the reputation of services in rural India [63]. Informal peer to peer education and review sessions were credited with improving staff performance and motivation in multiple studies [25,33,60] [35] [44], in preference to more traditional didactic approaches.

Significant gains also occurred by improving communication between health facilities and the communities they served. Community mobilisation and birth preparedness programs were credited [58,59] with increasing EMOC usage by prompting referral plans. The introduction of community feedback mechanisms led to improved utilisation and mortality outcomes in at least one study [28]. A mandatory blood donation policy was successfully introduced at one facility following significant community consultation [39]. The success of these small scale approaches was strongly dependent on fostering local leadership and staff morale [29,52]. 

### The role of context

As the examples above demonstrate, local context is fundamental to the success or failure of quality improvement strategies. The limited impact of IMCI on under-five mortality due to pneumonia in Bangladesh as compared to other countries [36,39] was attributed to a failure to adapt IMCI guidelines for high levels of antibiotic resistance. Similarly, training in clean delivery had no impact on maternal outcomes in areas where lack of clean water and sanitation resulted in almost immediate recontamination of cleansed items [42]. 

## Discussion

There is a disappointingly low level of evidence linking quality improvement strategies to improved MNCH outcomes. Methodologically, the quality of evidence was poor, and dominated by studies of individual facilities. Despite the thousands of references screened few studies of strategy implementation provided quantitative measurement of their impact; less than fifty contained sufficient information to determine that increases in quality occurred in conjunction with improved outcomes. Remarkably, over forty studies excluded from this review reported on quality improvement strategies but did not include any quantifiable measure to determine if quality had improved. This occurred despite our acceptance of definitions extending beyond traditional measures of quality, such as clinical practice. Consequently it is very difficult to justify wider implementation of particular strategies based on this evidence.

The heavy reliance on service utilisation as an indicator of the success of the strategy is also problematic. Health service usage is known to be heavily influenced by a number of factors separate from service quality [70-72]. The implicit assumption that quality improvements will result in increased utilisation, thus leading to improved outcomes, is not supported by the evidence. Utilisation appeared to be more strongly associated with patient perceptions than technical performance. Without appreciating this disconnect between utilisation and clinical quality it is possible that strategies aimed at improving health outcomes may in fact worsen existing issues. 

Another point of concern is the high probability of publication bias affecting our results. At least one positive outcome, either in terms of outcomes or quality improvement, was reported in all studies. It is possible that initial stages of the review excluded studies containing relevant information that omitted results due to lack of impact. It is more likely, however, that quality improvement studies failed to publish if there were no positive findings. This is problematic as even negative findings have great potential to guide future research given the limited nature of available evidence. 

The absence of strategies addressing elements such as distribution systems, public-private partnership and equity in service provision suggests another avenue for exploration. Existing evidence is dominated by large, multi-country initiatives such as IMCI, PMM and AMDD. These programs tended to have a heavy institutional focus, targeting physical determinants of quality and immediate factors affecting service delivery. While none of these initiatives can be considered complete failures, they were not as effective as expected. The variation in results in different contexts, combined with the oft cited need for additional policy support, suggests that there is scope for future studies to the effect of high level policy interventions on quality of health services. 

This review is limited by a number of factors. We only examined MNCH specific strategies, which may exclude relevant findings from related areas of health. Studies documented in places other than the academic literature were not included. This, combined with the relatively low methodological quality of the papers reviewed and the strong likelihood of positive reporting bias limits the strength of our conclusions. Finally, our strict inclusion criteria may have led to exclusion of studies examining quality of MNCH care, but for which there is no evidence regarding the pathway between strategies, quality and health system outcomes.

## Conclusions

While improving quality is not easy, it is a worthwhile goal. The existing evidence for the impact of quality improvement strategies on MNCH outcomes in low and middle income settings is limited; however the inclusion of more appropriate indicators in future studies would greatly expand our understanding of the effect of different strategies. Additional research into non-facility determinants of health service quality such as health policy, supply distribution, community acceptability and equity of care may also prove to be beneficial. Without such research quality improvement initiatives may falter, and without quality, improved health will remain a distant dream.

## Supporting Information

Checklist S1 **PRISMA checklist.**
(DOC)

Table S1 **Search terms used in initial search of MEDLINE, SCOPUS and CINAHL Databases.**
(DOCX)

Table S2 **Summary of Studies included in the Review, by Type of Strategy.**
(DOCX)
