# Supplementary material for: Do Strategies to Improve Quality of Maternal and Child Health Care in Lower and Middle Income Countries Lead to Improved Outcomes? A Review of the Evidence
Source: PLoS One. 2013 Dec 9;8(12):e83070. doi: 10.1371/journal.pone.0083070 (PMC3857295; doi:10.1371/journal.pone.0083070)
Supplement: Table S1 — Search terms used in initial search of MEDLINE, SCOPUS and CINAHL Databases. (DOCX) [file pone.0083070.s002.docx]

**Table S1: Search terms used in initial search of MEDLINE, SCOPUS and CINAHL Databases**

| a. | OR | (Afghanistan or Albania or Algeria or Angola or Antigua or Barbuda or Argentina or Armenia or Azerbaijan or Bangladesh or Belarus or Belize or Benin or Bhutan or Bolivia or Bosnia or Herzegovina or Botswana or Brazil or Bulgaria or Burkina Faso or Burundi or Cambodia or Cameroon or Cape Verde or Central African Republic or Chad or Chile or China or Colombia or Comoros or Congo or Costa Rica or Cote d'Ivoire or Cuba or Djibouti or Dominica or Dominican Republic or Ecuador or Egypt or El Salvador or Eritrea or Ethiopia or Fiji or Gabon or Gambia or Georgia or Ghana or Grenada or Guatemala or Guinea or Bissau or Guyana or Haiti or Honduras or India or Indonesia or Iran or Iraq or Jamaica or Jordan or Kazakhstan or Kenya or Kiribati or Korea or Kosovo or Kyrgyz* or Lao or Laos or Latvia or Lebanon or Lesotho or Liberia or Libya or Lithuania or Macedonia or Madagascar or Malawi or Malaysia or Maldives or Mali or Marshall Islands or Mauritania or Mauritius or Mayotte or Mexico or Micronesia or Moldova or Mongolia or Montenegro or Morocco or Mozambique or Myanmar or Namibia or Nepal or Nicaragua or Niger or Nigeria or Pakistan or Palau or Panama or Papua New Guinea or Paraguay or Peru or Philippines or Romania or Russia or Rwanda or Samoa or Sao Tome or Principe or Senegal or Serbia or Seychelles or Sierra Leone or Solomon Islands or Somalia or South Africa or Sri Lanka or Kitts or Nevis or Lucia or Vincent or Grenadines or Sudan or Suriname or Swaziland or Syria or Tajikistan or Tanzania or Thailand or Timor-Leste or East Timor or Togo or Tonga or Tunisia or Turkey or Turkmenistan or Tuvalu or Uganda or Ukraine or Uruguay or Uzbekistan or Vanuatu or Venezuela or Vietnam or West Bank or Gaza or Yemen or Zambia or Zimbabwe) |
| --- | --- | --- |
|  |  | Developing Countries/ |
| b. |  | ((Child* or Maternal or Neonat* or Infant* or pregnan* or obstetric) and (illness or care or mortality or deaths or health)) |
| c. | OR | ((Quality or performance or effectiveness) and (care or improvement*or increase* or service* or indicator*)) |
|  |  | Quality Assurance, Health Care/ or Quality Indicators, Health Care/ or Quality Improvement/ or Quality Control/ or "Quality of Health Care"/ |
| Final search terms combined search terms a, b and c using “AND” operator  Results limited to publication date post 1990 | | |
