# Supplementary material for: Do Strategies to Improve Quality of Maternal and Child Health Care in Lower and Middle Income Countries Lead to Improved Outcomes? A Review of the Evidence
Source: PLoS One. 2013 Dec 9;8(12):e83070. doi: 10.1371/journal.pone.0083070 (PMC3857295; doi:10.1371/journal.pone.0083070)
Supplement: Table S2 — Summary of Studies included in the Review, by Type of Strategy. (DOCX) [file pone.0083070.s003.docx]

**Table S2: Summary of Studies included in the Review, by Type of Strategy**

| **Primary Author (Year)** | **Intervention** | **Setting** | **Study Type** | **Study Quality** | **Quality Measures** | **Outcome Measures** | **Reported Effect on Outcome** | **Results** |
| --- | --- | --- | --- | --- | --- | --- | --- | --- |
| **Audit/Feedback/Guidelines** | | | | | | | | |
| Aghlmand (2008)[[26](#_ENREF_26)] | Introduction of new clinical practice guidelines for maternal care, with training courses and quality improvement workshops | 1 referral Hospital, Iran | Observational | Moderate | Clinical Practice | Satisfaction | *Positive* | Use of appropriate procedures increased from 0% to 78%. Significant increase in patient satisfaction for sixteen of the twenty needs and requirements. The proportion of caesarean deliveries decreased from 42% to 30% (RR 3.55 for deliveries where new guidelines not followed). |
| Björkman (2009)[[28](#_ENREF_28)] | Feedback to community about performance of primary health facilities via report card dissemination | 50 Rural Public Dispensaries, Uganda | Cluster RCT | High | Health services provision | Utilisation | *Positive* | Immunization of children, waiting time, examination procedures, and absenteeism improved significantly in the treatment communities. 0.14 z-score increase and 33 percent reduction in under-5 mortality in the treatment communities.  Utilization for general outpatient services 20 percent higher in the treatment compared to the control facilities and the overall effect across a set of utilization measures was large and significantly positive. |
|  |  |  |  |  |  | Mortality | *Positive* |  |
|  |  |  |  |  |  | Morbidity | *Positive* |  |
|  |  |  |  |  |  | Satisfaction | *Positive* |  |
| Bradley (2005)[[30](#_ENREF_30)] | Participatory quality improvement program using self-administered guides with trigger questions | 16 Rural/Peri Urban Health centres, Kenya and Guinea | RCT | High | Clinical Practice | Satisfaction | *Positive* | Statistically significant differences in most measures of quality for child consultations -no significant difference in diagnosing or treating sick children at intervention versus control sites. Oral rehydration salts (ORS) rarely used at either. 80% of clients in intervention sites said that they were better than they had been the year before, compared with 26.9% of clients in control sites (P < 0.01). |
| Chan (2010) [[29](#_ENREF_29)] | Dissemination and display of report cards on quality at facility | 8 temporary clinics for displaced populations, Aceh, Indonesia | Observational | Moderate | Health services provision | Utilisation | *Positive* | Percentage of residents receiving outreach activities increased from 14% to 85%, availability of essential drugs increased from 49% to 59%. Community satisfaction increased from 68% to 84%, up to date immunisation for children under-5 increased from 6% to 40%. |
|  |  |  |  |  |  | Satisfaction | *Positive* |  |
| Chowdhury (2008)[[27](#_ENREF_27)] | Introduction of new referral guidelines | 10 Primary Health facilities, Matlab Subdistrict, Bangladesh | Observational | High | Clinical Practice | Utilisation | *Positive* | 36% of severe pneumonia cases appropriately managed at baseline, increasing to 90% after revision. Proportion of referred cases receiving one does of injectable antibiotics prior to referral decreased from 76% to 35%. Overall visits by sick children increased 75%, and 97% for pneumonia visits. Case fatality rates did not significantly change (1.1% to 0.6% p=0.39). All cause mortality significantly declined (by 41%), pneumonia deaths did not. Ongoing IMCI efforts in area make attribution of effect to guideline revision difficult. |
|  |  |  |  |  |  | Mortality | *Neutral* |  |
| Figueras (2008)[[31](#_ENREF_31)] | Quality improvement program involving audits and creation of guidelines | 17 hospitals, Peru, Nicaragua, Argentina, Dominican Republic and Guatemala | Observational | Moderate | Clinical Practice | Morbidity | *Positive* | Administration of oxytocin increased significantly (p<0.05) from 71% to 85.9%, cord clamping from 68.2% to 87.5%, controlled cord traction from 43.2% to 73% and uterine massage from 42.5% to 75.7%. Full active management increased from 26.3% to 58.7%. Incidence of PPH decreased from 12.7% to 5% (P<0.05). |
| Hermida (2002)[[32](#_ENREF_32)] | Quality assurance program involving formation of teams to address specific issues | 8 small highland hospitals, Ecuador | RCT | Low | Clinical Practice | Utilisation | *Positive* | All indicators of clinical quality improved in intervention sites, final levels were significantly greater than in controls with exception of pneumonia treatment. Final levels higher for input indicators (such as supply of drugs and equipment) rather than process indicators (such as complete ANC and use of partograph).  Maternal knowledge of danger signs and client satisfaction remained similar between intervention and controls. ANC consultations increased by 18.5% in intervention sites compared to 11.2% in controls. Outpatient visits for sick children increased by 21.2% in intervention hospitals and 34.4% in control. No increase in deliveries for either group. |
|  |  |  |  |  |  | Satisfaction | *Neutral* |  |
| Kongnyuy (2008)[[22](#_ENREF_22)] | Maternal death review | 3 districts, Central Region, Malawi | Observational | High | Case fatality rate | Utilisation | *Mixed* | Case fatality rates decreased from 3.7% to 1.5%. Maternal mortality dropped from 250 per 100000 women to 182. Met need for EMOC rose from 15.2% to 18.8%. Institutional delivery and C-section rate did not change significantly. |
|  |  |  |  |  |  | Mortality | *Positive* |  |
| Kongnyuy (2009)[[23](#_ENREF_23)] | Audit of women friendly behaviours during delivery | 3 districts, Central Region, Malawi | Observational | High | Satisfaction | Utilisation | *Positive* | Significantly improved client reception, privacy using curtains of screens, support by a companion during labour, provider-client communication, client choice of birthing position and overall client satisfaction. Number of facility deliveries increased by 31%. |
| Pattinson (2006)[[33](#_ENREF_33)] | New guideline for management of Severe Acute Maternal Morbidity | 4 hospitals, Pretoria, South Africa | Observational | High | Clinical Practice | Utilisation | *Positive* | Significant decline in proportion of cases citing lack of facilities (22.1% to 7.1%) and lack of blood transfusion (3.1% to 0.4%) as contributing factors. Substandard management fell significantly from 26% to 9.7%.  Total number of births increased by 27%, and prevalence of critically ill pregnant women also increased. MMR reduced, from 133.2/100 000 live births in 1997/1998 to 104.4/100 000 in 2002-2004. Mortality index fell from 15.9% in 1997/1998 to 10.3% in 2002/2004. |
|  |  |  |  |  |  | Mortality | *Positive* |  |
| Srofenyoh (2012)[[34](#_ENREF_34)] | Quality improvement program involving audits and creation of guidelines, | 1 referral hospital, Accra, Ghana | Observational | Moderate | Training score Clinical Practice  Case fatality rate | Mortality | *Positive* | CFR for Hypertension decreased from 3.1% in 2007 to 1.1% in 2009 (p<0.05) , CFR for haemorrhage decreased from 14.8% in 2007 to 1.9% in 2009. Neonatal resuscitation scores increased significantly, use of general anaesthesia in C-sections declined from 94% in 2006 to 11% in 2009. Reduction in maternal mortality from 473 to 328 deaths per 100 000 live births. 36% reduction in stillbirth and non-significant reduction in neonatal death. |
| Strand (2009)[[24](#_ENREF_24)] | Audit of emergency obstetric referrals followed by training and guidelines. | 3 facilities, Luanda, Angola | Observational | Low | Clinical Practice | Mortality | *Positive* | Time from admission to treatment decreased from mean 13.7hrs to 54-71 minutes after audit. Partogram quality improved from 11% and 0% rated good in referring and referral units respectively to 95% and 36%. CS rate increased from 13% to 30%, use of uterotonic treatment from 15% to 24%. CFR reduced from 17.8% to 0%. |
| Wilkinson (1997)[[25](#_ENREF_25)] | Audit of maternal and perinatal procedures | 1 rural district, KwaZulu-Natal Province, South Aftrica | Observational | Moderate | Avoidable Mortality | Utilisation | *Positive* | Average monthly number of deliveries increased from 325 in 1991 to 424 in 1995 (31% increase). Perinatal mortality rate increased from 27/1000 in 1991 to 42 in 1992. Thereafter it steadily declined to 26/1000 in 1995. Proportion of avoidable deaths followed different pattern and fell from 19% in 1991 to 0% in 1995. |
| Youngleson (2010)[[35](#_ENREF_35)] | Quality improvement process with minor increases in human resource | 1 urban sub-district, Cape Metro District, South Africa | Observational | Moderate | Health services provision | Morbidity | *Positive* | Provision of antenatal AZT increased from 72% to 89%, NVP and AZT at delivery increased from 74% to 86% and 43% to 84%. Infant follow-up increased from 79% to 95%. Perinatal HIV transmission decreased in Eastern sub-district from 7.6% to 5%, and in metropolitan Cape Town from 4.2% to 3.8%. |
| ***IMCI*** | | | | | | | | |
| el Arifeen (2004) (2009)[[36](#_ENREF_36),[37](#_ENREF_37)] | IMCI with training, additional supervision, supply tracking system, basic job aids (scales, thermometer etc), referral guidelines. | 20 Primary Health facilities, Matlab Subdistrict, Bangladesh | Cluster RCT | High | Clinical Practice | Utilisation | *Positive* | Mean index of correct treatment and counselling in IMCI facilities rose from 8% to 54% between 2001 and 2004. Levels remained between 64-68% thereafter. Did not improve significantly in control. Proportion of ill children taken to appropriate provider increased in IMCI areas from 9% in 2000 to 24% in 2007, compared to controls where it remained between 4-8%.  Under 5 mortality rates fell from 70·0 and 65·6 per 1000 live births (p=0·32) in intervention and comparison areas to 49·3 and 50·5 per 1000 live births (p=0·75). After adjustment for baseline rates, the difference corresponded to 3·3 (95% CI –3·4 to 10·0) fewer deaths per 1000 live births in IMCI areas (p=0·31). No evidence of significant accelerations in reductions of mortality. 12% decrease in stunting in children aged 24–59 months in intervention areas compared to 5% in comparison areas. Wasting dropped substantially in both areas, but differences between areas were not significant. |
|  |  |  |  |  |  | Mortality | *Neutral* |  |
|  |  |  |  |  |  | Morbidity | *Neutral* |  |
| Bryce (2010)[[39](#_ENREF_39)] | EPI+, ANC+, IMCI+ | Intervention districts in Benin, Ghana and Mali | Observational with comparison group | High | Health services provision | Mortality | *Neutral** | A few interventions for which coverage of health interventions accelerated and some important interventions for which coverage decelerated. Most gains in focus districts occurred for outreach interventions (vaccination, ITNs).IMCI fared particularly badly. No significant differences in malnutrition that could be attributed to Program. Mortality decreased in all focus districts but did not differ significantly from comparison areas. |
|  |  |  |  |  |  | Morbidity | *Neutral** |  |
| Rowe (2011)[[38](#_ENREF_38)] | IMCI with community ITN promotion | 4 departments (with ~100 facilities each), Benin | Observational with comparison group | Moderate | Health services provision Clinical Practice | Utilisation | *Positive* | Correct management of potentially life threatening illness increased from 25.5% to 44.4% (p<0.001). Correct treatment of severe illness increased from 29% to 56.6%. Proportion of facilities with all key drugs in stock increased from 37.7% to 52%. Utilisation of facilities for childhood illness increased 18.4% from 304 visits per weekday to 360 per weekday. Mortality rate decreased from 105.9/1000 live births to 92.2/1000 compared to 106.8/1000 to 108.3/1000 in comparison area. Difference was significant. Control group was contaminated, with ITN promotion occurring in both districts. Similar changes in ITN use noted, leading authors to conclude that mortality reduction was due to IMCI. |
|  |  |  |  |  |  | Mortality | *Positive* |  |
| **Training** |  |  |  |  |  |  |  |  |
| Goodburn (2000)[[42](#_ENREF_42)] | Training of TBAs in clean delivery | Rural Bangladesh | Observational | High | Clinical Practice | Morbidity | *Neutral* | Trained TBAs twice as likely as untrained TBAs to perform clean delivery (45% vs. 19.3%) however also more likely to insert hands into vagina. No significant differences in regards to maternal infection. No association between maternal infection and clean delivery. Maternal infection significantly associated with existing RTI, insertion of hand into vagina, malnutrition and non-primagravidity. |
| Ohnishi (2007) [[40](#_ENREF_40)] | Training in ANC provision | Rural Caazapa region, Paraguay | Observational | Low | Training score | Utilisation | *Positive* | Mean pre-test score was 41 (of 100), increased to 60.1 - difference was significant (p<0.001). Average number of ANC visits per pregnancy increased from 2.2 in 1996 to 3.4 in 1998. |
| O'Rourke (1995)[[46](#_ENREF_46)] | Training of staff to improve treatment towards TBA referrals | 1 referral hospital, rural Guatemala | Observational | High | Satisfaction | Utilisation | *Positive* | Prior to training period 79% of TBA referred women indicated they would use hospital again, increased to 94% post training (significant). Time between admission and treatment changed, % of deliveries with no wait increased from 8.11% to 31.09%, however proportion waiting over 1 hr remained the same (47.3% - 46.92%). Mean number of referrals increased by over 200%, significantly associated with post-training period. No statistically significant effect of hospital training program on perinatal mortality. |
|  |  |  |  |  |  | Mortality | *Neutral* |  |
| Ouma (2010)[[41](#_ENREF_41)] | Focused ANC training | 2 districts, Nyanza province, Kenya | Observational with comparison group | High | Clinical Practice | Utilisation | *Neutral* | Provision of Iron, folic acid and IPT increased, but other services such as counselling did not change. ANC usage remained the same. 17% of women in intervention area rated ANC as very satisfactory versus 6.5% in control. |
|  |  |  |  |  |  | Satisfaction | *Positive* |  |
| Robinson (2001)[[44](#_ENREF_44)] | Peer to peer training in immunisation | 15 rural Health Centres, Maluku Province, Indonesia | Observational | Moderate | Health services provision | Utilisation | *Positive* | Average number of immunisation practices performed correctly increased from 7.4 (of 12) pre training to 10.2 post training (30% increase). Coverage of DPT1, Polio3 and Measles all significantly improved in intervention facilities while control facilities remained the same. Composite immunisation coverage rose from 41% to 68% in interventions compared to 58-60% in controls. Excluding two facilities which had major transport issues during the period the final composite coverage in intervention facilities was even higher at 75%. Further limiting to only facilities in which trained staff were not reassigned led to change in coverage from 46% to 84% post training. |
| Senarath(2007)[[43](#_ENREF_43)] | Training in Essential Neonatal Care | 5 hospitals, North Western Province, Sri Lanka | RCT | High | Clinical Practice | Utilisation | *Positive* | Significant improvements in practices of cleanliness at delivery, thermal protection, preparedness for resuscitation and neonatal assessment in intervention group. Proportion of any undesirable health even declined from 14.3% to 9.4% (p= >.05) in intervention and from 9% to 7.6% in control. Significant increase in breastfeeding within 30 min from 45.3% to 70.4%. |
|  |  |  |  |  |  | Morbidity | *Positive* |  |
| Uskun (2008)[[45](#_ENREF_45)] | Formal training in immunisation | 86 facilities, Isparta City, Turkey | Observational | Low | Training score | Utilisation | *Positive* | Mean pre-test score was 21.5 (of 40), increased to 36.7 - difference was significant (p<0.001). Significant increases in vaccination coverage before and after training period. |
| ***Financial*** | | | | | | | | |
| Basinga (2011)[[47](#_ENREF_47)] | Pay for Performance (P4P) - additional money given to facility based on coverage of key indicators (e.g. $ per skilled delivery) and quality index (used to adjust payments to reward good quality) | 116 Health Facilities, Rwanda | Cluster RCT | High | Health services provision Clinical Practice | Utilisation | *Mixed* | Mixed results - increased use and quality of some services but not others. Probably related to structure of incentives - provider controlled interventions such as 2TT and quality of ANC increased, but # ANC visits requiring user decision to come to facility did not. Institutional deliveries increased significantly but no impact on full vaccination. |
| Renaudin (2007)[[21](#_ENREF_21)] | Obstetric risk insurance - enrolled patients entitled to full maternity care with no additional user fees, profits used for drugs and financial incentives. | Urban area, Nouakchott City, Mauritania | Observational | Moderate | Clinical Practice | Utilisation | *Positive** | Average number of pre-natal consultations was 2.6 for enrolled women versus 1.7 for non-enrolled. 78% versus 25% outside received an ultrasound. Post-natal consultation rate is 81% vs. 50%.  Quality of care did not improve, with offer of preventive measures during antenatal and post-natal visits declining. Only 68% of women received the standardized prophylactic treatment for anaemia and 31% for malaria. Urine tests were not performed in 67% of consultations in 2005. |
| Soeters (2011)[[48](#_ENREF_48)] | Performance based financing (contracting to do services, bonus if quality score was good, ability to raise/lower user fees - limited details on how exactly the system was run) | 4 districts, South Kivu Province, Democratic Republic of Congo | Observational with comparison group | Moderate | Satisfaction | Utilisation | *Positive* | Significant (p<0.05) improvements in patient perceived quality and availability of drugs, as well as professional quality scores in intervention as compared to control sites. Knowledge of HIV/AIDS was significantly better in intervention, but childbirth in a health facility was much greater in the control districts (p<0.05). General use of health facilities was significantly greater in intervention facilities (p<0.10). Use of ITN, FP, sanitary latrine, ANC and Vaccination were generally greater in intervention but not significantly so. Annual household per capita health expenditure increased 45% in intervention districts and decreased 18% in control. However expenditure for poorest 25% of sample decreased 14% in intervention and increased 67% in control. Disease episodes not treated due to shortage of money decreased by 15% in intervention districts compared to 5% in control (significant p<0.10). |
|  |  |  |  |  |  | Out of Pocket Expenses | *Mixed* |  |
| ***Complex*** | | | | | | | | |
| Barua (2003) [[63](#_ENREF_63)] | ANC improvements - ensuring staff had necessary equipment, increased monitoring and supervision, focused training, re-organisation of outreach clinics and scheduling. | Rural District, Maharashtra State, India | Observational | Moderate | Clinical Practice | Utilisation | *Positive* | Quality of care increased, >60% of women all ANC components. Blood pressure and urine testing increased significantly (from 50 to 94% for BP and 46-89% for urine). Attendance at clinics rose by 30% (from average for 4-5 women per camp to 15-20 women per camp) |
| Fort (2011) [[64](#_ENREF_64)] | New primary health care model. Involved meetings and training, clinical guidelines, monitoring and supervision, active case-finding. | 2 rural municipalities, Guatemala | Observational | High | Health services provision Clinical Practice | Utilisation | *Positive* | Proportion of women with at least one PNC visit increased from 88.8% to 100% in one site and 54% to 98% in the other. Mean number of days before first PNC visit fell (5.6 - 3.3 in site 1, 13.3-4.1 in site 2). Correct treatment for infant pneumonia increased from 49.4-61.9% to 94-96.3%, for 1-4yr pneumonia rose from 28-42.4% to 92.7-97.8%. Correct treatment of tonsillitis increased 55-84% to 96.6-97.2%. Complete vaccination for children 12-23 months rose from 48.1-44.8% to 96.1-92.7%. Full tetanus immunisation for women 15-49 increased from 13.7-7.9% to 62-54.7%. Any ANC rose from 64.5-40.5% to 98.4-93.9%. ANC 3+ increased from 27.2-16% to 71.9-62.5%. Mean number of clinic visits per family member per year increased from 0.89 to 14 in site 1 and 0.32-1.08 in site 2. Significant (99%) reduction in percentage of families with no clinic visits each year. |
| Ifenne (1997) [[49](#_ENREF_49)] | Facility upgrades, staff training, blood bank and blood donation policy, emergency drug pack system | 1 tertiary hospital, Zaria, Nigeria | Observational | Moderate | Case fatality rate  Clinical Practice | Utilisation | *Negative* | CFR declined from 14% to 11%, mean admission to treatment interval for obstetric complications fell from 3.7hrs to 1.6hrs, and % treated within 30 min increased from 39% to 87%. Utilisation fell substantially, with the total number of deliveries falling by 60% and the number of complicated deliveries falling 80%. |
| Kayongo (2006) [[55](#_ENREF_55)] | Training, creation of guidelines, improved data management, audits, supervision, facility and equipment renovation/maintenance. | 5 health facilities, Ayacucho, Peru | Observational | Moderate | Case fatality rate  Health services provision | Utilisation | *Mixed* | Case fatality rates decreased from 1.7% to 0.1%. Met need for EMOC rose from 30.4% to 83.9%. C-section rate rose from 3.9% to 6%. Proportion of births in project facilities fluctuated, but did not change significantly. |
| Kayongo (2006) [[54](#_ENREF_54)] | Training, creation of guidelines, improved data management, audits, supervision, facility and equipment renovation/maintenance. | 1 large referral hospital, Gitarama, Rwanda | Observational | Moderate | Case fatality rate  Health services provision | Utilisation | *Mixed* | Case fatality rates decreased from 2.2% to 1.3%. Met need for EMOC rose from 16.2% to 24.8%. C-section rate rose from 1.9% to 3.2%. Proportion of births in EMOC facilities fluctuated, but did not change significantly. |
| Kayongo (2006)[[56](#_ENREF_56)] | Training, creation of guidelines, improved data management, audits, supervision, facility and equipment renovation/maintenance. | 10 hospitals in Tanzania, Rwanda and Ethiopia | Observational | Moderate | Case fatality rate  Health services provision | Utilisation | *Mixed* | Case fatality rates decreased from 3% to 1.9% in Tanzania, 10.4% to 5.2% in Ethiopia and 2% to 0.9% in Rwanda. Met need for EMOC rose from 9.1% to 19.3% in Tanzania, 2% to 4.5% in Ethiopia and 16.2% to 24.6% in Rwanda. C-section rate rose from 1.2% to 1.8% in Tanzania, 0.2 - 0.4% in Ethiopia and 1.8-2.9% in Rwanda. Proportion of births in EMOC facilities fluctuated, but did not change significantly in Rwanda and Ethiopia, rose from 13.1 to 17.9% in Tanzania. |
| Leigh (1997)[[50](#_ENREF_50)] | Facility upgrades, drugs and supplies, staff training, hiring of trained staff, policy of treatment before payment and (briefly) staff incentives. | 1 referral hospital, Makeni, Sierra Leone | Observational | Moderate | Case fatality rate | Utilisation | *Mixed* | CFR fell from 32% in 1990 to 5% in 1995. Utilisation (both normal and complicated cases) rose until 1993, before falling slightly (1995 levels similar to 1992). Disruption due to war likely. Caesarean sections rose steadily as did total obstetric procedures. Very high uptake of abortion related procedures (from 22 at baseline to 444 in 1995). |
| McQuestion (2006) [[62](#_ENREF_62)] | EMOC upgrading - setting up community health committees, facility improvements, retraining, incorporation of local birthing practices into clinical protocols, auto evaluation (continuous quality care monitoring) | Multiple facilities, Ayacucho, Peru | Observational | High | Health services provision Clinical Practice | Utilisation | *Neutral* | Program improved the quality of care in intervention areas but did not directly increase the probability of delivery in EMOC facilities. Only behavioural impact noted was that reducing out-of pocket costs (via social insurance program unrelated to quality improvements) increases EMOC utilization. |
| Mekbib (2003) [[61](#_ENREF_61)] | Facility upgrades, drugs and supplies, staff training, record keeping, blood supply. | Two health centres, West Showa Zone, Ethiopia | Observational | Moderate | Case fatality rate | Utilisation | *Positive* | CFR fell from 7.2% in 1999 to 4.6% in 2001. Utilisation of facilities rose from 6.08% to 7.62%, however much of this rise was complicated deliveries. C-sections increased from 3.8% of all births in 1998 to 17.3% in 2001. |
| Olukoya (1997) [[51](#_ENREF_51)] | Facility upgrades, staff training, record keeping, revolving drug fund, blood bank, maternal death review, community mobilisation | 1 referral hospital, Rural Ogun, Nigeria | Observational | Moderate | Case fatality rate | Utilisation | *Mixed* | CFR remained constant at 7-8%. Utilisation decreased from 1992 until 1994 before recovering in 1995. Similar pattern seen for complications. |
| Otchere (2007) [[58](#_ENREF_58)] | Training, creation of guidelines, improved data management, audits, supervision, facility and equipment renovation/maintenance. | 2 district hospitals, Sikasso region, Mali | Observational | Moderate | Case fatality rate  Health services provision | Utilisation | *Mixed* | Case fatality rates decreased from 7.1% to 2.2% and 8.2% to 5.1%. Met need for EMOC rose from 8.8% to 15% and 6.4% to 15.3%. C-section rate rose from 0.8% to 1.2% and 0.6 to 0.8%. Proportion of births in project facilities fluctuated, but did not change significantly. |
| Otchere (2007) [[57](#_ENREF_57)] | Facility upgrades, equipment, training, strengthened referral (telephones), supervision, quality improvement tools, IEC | 2 district hospitals, Than Hoa and Quan Tri Provinces, Vietnam | Observational | Moderate | Case fatality rate  Health services provision | Utilisation | *Positive* | Case fatality rates did not change significantly. Met need for EMOC rose from 16.1% to 87% in Hai Lang district and 17.2-54.3% in Hoa Hang. C-section rate rose from 4.9% to 6.8%. Proportion of births in EMOC facilities rose in both areas. |
| Oyesola (1997) [[52](#_ENREF_52)] | Contracting of specialists, training, use of emergency obstetric care boxes (drugs and equipment) | 1 referral hospital, Kebbi, Nigeria | Observational | Moderate | Case fatality rate | Utilisation | *Mixed* | CFR declined from 22% in 1990 to 5% in 1995, with declines occurring over virtually all complications. Utilisation of obstetric services fluctuated, rising until 1994 before dropping back to 1991 levels in 1995. Reduction in complicated cases is more pronounced. |
| Rana (2007) [[59](#_ENREF_59)] | Infrastructure and equipment upgrades, monitoring procedures, filling vacancies, team building, training, quality improvement program | 4 rural districts, Nepal | Observational | Moderate | Case fatality rate  Health services provision | Utilisation | *Positive* | Case fatality rate declined from 2.7 to 0.3. Met need for EMOC increased from 1.9% to 16.9% (utilisation increased from 1971 births in 2000 to 4623 in 2004). C-sections increased from 0.4% to 0.7% |
| Sabitu (1997) [[53](#_ENREF_53)] | Revolving drug fund, midwife training and ambulance provision | 1 referral hospital, Zaria, Nigeria | Observational | Moderate | Health services provision Clinical Practice | Utilisation | *Mixed* | Drug availability increased from 10% to 80%, mean admission to treatment interval dropped from 9.5 hours to 1.5 hours. However number of women with complications admitted fell from 85 to 28 (-67%), despite 500% increase in normal deliveries. Referral to tertiary facilities increased from 4 to 17, and mean time between decision to refer and departure dropped from 48hrs to 45mins. |
| Santos (2006) [[60](#_ENREF_60)] | Infrastructure, human resource development, transportation and communication systems, supportive supervision, logistics for supplies, equipment and drugs, record keeping, and quality improvement techniques | 23 facilities, Sofala province, Mozambique | Observational | Moderate | Case fatality rate  Health services provision | Utilisation | *Positive* | Case fatality rate declined from 2.9 to 1.6. Met need for EMOC increased from 11.3% to 32.8% (utilisation increased from 8262 births in 2000 to 26257 in 2005). C-sections increased from 1.1% to 1.8% |
| ***Other*** | | | | | | | | |
| Jafari (2010) [[66](#_ENREF_66),[67](#_ENREF_67)] | Group ANC sessions | 14 urban public health centres, Zanjan, Iran | RCT | High | Satisfaction | Utilisation | *Positive* | Mean patient satisfaction score was 3.8 for women in group sessions versus 3.1 in individual care. Initiation of ANC was the same between groups, but 37.3% of women in individual care received the full number of ANC visits compared to. 70.3% in group sessions. Significantly greater birth weight and use of supplements and postpartum contraception in group ANC. Also significantly better breastfeeding rates at 2 months. Time to diagnosis for UTI and RTI also significantly lower. |
|  |  |  |  |  |  | Morbidity | *Positive* |  |
| Marin (2011) [[65](#_ENREF_65)] | Provision of personalised ANC (named provider, increased follow-up) | La Plata district, Buenos Aires, Argentina | Observational | High | Health Services Provision | Utilisation | *Positive* | Average number of ANC visits increased from 3.04 to 3.93, average number of ANC visits for High Risk pregnancy increased from 4.23 to 6.78. Proportion of pregnancies detected before 14 weeks increased significantly from 31.2% to 43.7%. <16 weeks rose from 44.1% to 60.3%. No significant change in proportion <20 weeks. Tetanus vaccination at time of delivery rose from 60.8% to 100%.  Number of pregnant women rose dramatically from 1569 in 2006 to 6668 in 2009 (p<0.00001). Percentage of LBW newborns declined from 30.85% to 6.88% (p<0.001). Maternal deaths within the program declined from 7 in 2006 to 0 in 2009. Maternal mortality rate for the region fell from 6.9 to 3.16 (both changes significant P=0.001). Infant mortality fell from 13.7/1000 to 11.8 (p = 0.039). |
|  |  |  |  |  |  | Mortality | *Positive* |  |
|  |  |  |  |  |  | Morbidity | *Positive* |  |

**Study did not report improvement in all quality measures.*

**Appendix A References**

1. Boschi-Pinto C, Young M, Black RE (2010) The child health epidemiology reference group reviews of the effectiveness of interventions to reduce maternal, neonatal and child mortality. International Journal of Epidemiology 39: i3-i6.

2. Campbell OM, Graham WJ (2006) Strategies for reducing maternal mortality: getting on with what works. The Lancet 368: 1284-1299.

3. Darmstadt GL, Bhutta ZA, Cousens S, Adam T, Walker N, et al. (2005) Evidence-based, cost-effective interventions: how many newborn babies can we save? The Lancet 365: 977-988.

4. Graham WJ, Varghese B (2012) Quality, quality, quality: gaps in the continuum of care. The Lancet 379: e5-e6.

5. Althabe F, Bergel E, Cafferata ML, Gibbons L, Ciapponi A, et al. (2008) Strategies for improving the quality of health care in maternal and child health in low- and middle-income countries: An overview of systematic reviews. Paediatric and Perinatal Epidemiology 22: 42-60.

6. Raven J, Hofman J, Adegoke A, van den Broek N (2011) Methodology and tools for quality improvement in maternal and newborn health care. International Journal of Gynaecology & Obstetrics 114: 4-9.

7. Pirkle CM, Dumont A, Zunzunegui M-V (2011) Criterion-based clinical audit to assess quality of obstetrical care in low- and middle-income countries: a systematic review. International Journal for Quality in Health Care 23: 456-463.

8. Opiyo N, English M (2010) In-service training for health professionals to improve care of the seriously ill newborn or child in low and middle-income countries (Review). Cochrane Database of Systematic Reviews: CD007071.

9. van Lonkhuijzen L, Dijkman A, van Roosmalen J, Zeeman G, Scherpbier A (2010) A systematic review of the effectiveness of training in emergency obstetric care in low-resource environments. BJOG: An International Journal of Obstetrics & Gynaecology 117: 777-787.

10. Nyamtema AS, Urassa DP, van Roosmalen J (2011) Maternal health interventions in resource limited countries: A systematic review of packages, impacts and factors for change. BMC Pregnancy and Childbirth 11.

11. Haws RA, Thomas AL, Bhutta ZA, Darmstadt GL (2007) Impact of packaged interventions on neonatal health: a review of the evidence. Health Policy & Planning 22: 193-215.

12. Kongnyuy EJ, Van Den Broek N (2009) Audit for maternal and newborn health services in resource-poor countries. BJOG: An International Journal of Obstetrics and Gynaecology 116: 7-10.

13. Pattinson R, Kerber K, Waiswa P, Day LT, Mussell F, et al. (2009) Perinatal mortality audit: counting, accountability, and overcoming challenges in scaling up in low- and middle-income countries. International Journal of Gynaecology & Obstetrics 107 Suppl 1: S113-121, S121-112.

14. Peabody JW, Taguiwalo MM, Robalino DA, Frenk J (2006) Improving the Quality of Care in Developing Countries. In: Jamison DT, Breman JG, Measham AR, Alleyne G, Claeson M et al., editors. Disease Control Priorities in Developing Countries 2nd edition. Washington (DC): World Bank.

15. Entwistle V, Firnigl D, Ryan M, Francis J, Kinghorn P (2012) Which experiences of health care delivery matter to service users and why? A critical interpretive synthesis and conceptual map. Journal of Health Services Research and Policy 17: 70-78.

16. Raven JH, Tolhurst RJ, Tang S, van den Broek N (2011) What is quality in maternal and neonatal health care? Midwifery.

17. Wong ST, Yin D, Bhattacharyya O, Wang B, Liu L, et al. (2010) Developing a performance measurement framework and indicators for community health service facilities in urban China. BMC Family Practice 11.

18. Bravata DM, McDonald KM, Shojania KG, Sundaram V, Owens DK (2005) Challenges in Systematic Reviews: Synthesis of Topics Related to the Delivery, Organization, and Financing of Health Care. Annals of Internal Medicine 142: 1056-1065.

19. The Partnership for Maternal Newborn & Child Health (2011) A Global Review of the Key Interventions Related to Reproductive, Maternal, Newborn and Child Health (RMNCH). Geneva, Switzerland: PMNCH.

20. WHO (2000) World health report 2000: health systems: improving performance. World Health Organization.

21. Renaudin P, Prual A, Vangeenderhuysen C, Ould Abdelkader M, Ould Mohamed Vall M, et al. (2007) Ensuring financial access to emergency obstetric care: three years of experience with Obstetric Risk Insurance in Nouakchott, Mauritania. International Journal of Gynaecology & Obstetrics 99: 183-190.

22. Kongnyuy EJ, Leigh B, van den Broek N (2008) Effect of audit and feedback on the availability, utilisation and quality of emergency obstetric care in three districts in Malawi. Women & Birth: Journal of the Australian College of Midwives 21: 149-155.

23. Kongnyuy EJ, Mlava G, van den Broek N (2009) Criteria-based audit to improve women-friendly care in maternity units in Malawi. Journal of Obstetrics & Gynaecology Research 35: 483-489.

24. Strand RT, de Campos PA, Paulsson G, de Oliveira J, Bergström S (2009) Audit of referral of obstetric emergencies in Angola: a tool for assessing quality of care. African Journal of Reproductive Health 13: 75-85.

25. Wilkinson D (1997) Reducing perinatal mortality in developing countries. Health Policy and Planning 12: 161-165.

26. Aghlmand S, Akbari F, Lameei A, Mohammad K, Small R, et al. (2008) Developing evidence-based maternity care in Iran: a quality improvement study. BMC Pregnancy & Childbirth 8: 20.

27. Chowdhury EK, El Arifeen S, Rahman M, Hoque DE, Hossain MA, et al. (2008) Care at first-level facilities for children with severe pneumonia in Bangladesh: a cohort study. Lancet 372: 822-830.

28. Björkman M, Svensson J (2009) Power to the people: Evidence from a randomized field experiment on community-based monitoring in uganda. Quarterly Journal of Economics 124: 735-769.

29. Chan GJ, Parco KB, Sihombing ME, Tredwell SP, O'Rourke EJ (2010) Improving health services to displaced persons in Aceh, Indonesia: a balanced scorecard.[Erratum appears in Bull World Health Organ. 2010 Oct 1;88(10):796]. Bulletin of the World Health Organization 88: 709-712.

30. Bradley J, Igras S (2005) Improving the quality of child health services: participatory action by providers. International Journal for Quality in Health Care 17: 391-399.

31. Figueras A, Narváez E, Valsecia M, Vásquez S, Rojas G, et al. (2008) An education and motivation intervention to change clinical management of the third stage of labor - The GIRMMAHP Initiative. Birth 35: 283-290.

32. Hermida J, Robalino ME (2002) Increasing compliance with maternal and child care quality standards in Ecuador. International Journal for Quality in Health Care 14 Suppl 1: 25-34.

33. Pattinson RC, Macdonald AP, Backer F, Kleynhans M (2006) Effect of audit on critically ill pregnant women. Clinical Governance: An International Journal 11: 278-288.

34. Srofenyoh E, Ivester T, Engmann C, Olufolabi A, Bookman L, et al. (2012) Advancing obstetric and neonatal care in a regional hospital in Ghana via continuous quality improvement. International Journal of Gynecology and Obstetrics 116: 17-21.

35. Youngleson MS, Nkurunziza P, Jennings K, Arendse J, Mate KS, et al. (2010) Improving a mother to child HIV transmission programme through health system redesign: quality improvement, protocol adjustment and resource addition. PLoS ONE [Electronic Resource] 5: e13891.

36. el Arifeen S, Hoque DME, Akter T, Rahman M, Hoque ME, et al. (2009) Effect of the Integrated Management of Childhood Illness strategy on childhood mortality and nutrition in a rural area in Bangladesh: a cluster randomised trial. Lancet 374: 393-403.

37. el Arifeen S, Blum LS, Hoque DME, Chowdhury EK, Khan R, et al. (2004) Integrated Management of Childhood Illness (IMCI) in Bangladesh: early findings from a cluster-randomised study. Lancet 364: 1595-1602.

38. Rowe AK, Onikpo F, Lama M, Osterholt DM, Deming MS (2011) Impact of a malaria-control project in Benin that included the integrated management of childhood illness strategy. American Journal of Public Health 101: 2333-2341.

39. Bryce J, Gilroy K, Jones G, Hazel E, Black RE, et al. (2010) The Accelerated Child Survival and Development programme in west Africa: a retrospective evaluation. Lancet 375: 572-582.

40. Ohnishi M, Nakamura K, Takano T (2007) Training of healthcare personnel to improve performance of community-based antenatal care program. Advances in Health Sciences Education 12: 147-156.

41. Ouma PO, Van Eijk AM, Hamel MJ, Sikuku ES, Odhiambo FO, et al. (2010) Antenatal and delivery care in rural western Kenya: The effect of training health care workers to provide "focused antenatal care". Reproductive Health 7.

42. Goodburn EA, Chowdhury M, Gazi R, Marshall T, Graham W (2000) Training traditional birth attendants in clean delivery does not prevent postpartum infection. Health Policy & Planning 15: 394-399.

43. Senarath U, Fernando DN, Rodrigo I (2007) Effect of training for care providers on practice of essential newborn care in hospitals in Sri Lanka. JOGNN - Journal of Obstetric, Gynecologic, & Neonatal Nursing 36: 531-541.

44. Robinson JS, Burkhalter BR, Rasmussen B, Sugiono R (2001) Low-cost on-the-job peer training of nurses improved immunization coverage in Indonesia. Bulletin of the World Health Organization 79: 150-158.

45. Uskun E, Uskun SB, Uysalgenc M, Yagiz M (2008) Effectiveness of a training intervention on immunization to increase knowledge of primary healthcare workers and vaccination coverage rates. Public Health 122: 949-958.

46. O'Rourke K (1995) The effect of hospital staff training on management of obstetrical patients referred by traditional birth attendants. International Journal of Gynecology and Obstetrics 48: S95-S102.

47. Basinga P, Gertler PJ, Binagwaho A, Soucat AL, Sturdy J, et al. (2011) Effect on maternal and child health services in Rwanda of payment to primary health-care providers for performance: An impact evaluation. The Lancet 377: 1421-1428.

48. Soeters R, Peerenboom PB, Mushagalusa P, Kimanuka C (2011) Performance-Based Financing Experiment Improved Health Care In The Democratic Republic Of Congo. Health Affairs 30: 1518-1527.

49. Ifenne D, Essien E, Golji N, Sabitu K, Alti-Mu'azu M, et al. (1997) Improving the quality of obstetric care at the teaching hospital, Zaria, Nigeria. International Journal of Gynaecology & Obstetrics 59 Suppl 2: S37-46.

50. Leigh B, Kandeh HB, Kanu MS, Kuteh M, Palmer IS, et al. (1997) Improving emergency obstetric care at a district hospital, Makeni, Sierra Leone. The Freetown/Makeni PMM Team. International Journal of Gynaecology & Obstetrics 59 Suppl 2: S55-65.

51. Olukoya AA, Ogunyemi MA, Akitoye CO, Abudu O, Tijani MA, et al. (1997) Upgrading obstetric care at a secondary referral hospital, Ogun State, Nigeria. The Lagos PMM Team. International Journal of Gynaecology & Obstetrics 59 Suppl 2: S67-74.

52. Oyesola R, Shehu D, Lkeh AT, Maru I (1997) Improving emergency obstetric care at a state referral hospital, Kebbi State, Nigeria. International Journal of Gynecology and Obstetrics 59: S75-S81.

53. Sabitu K, Alti-Mu'azu M, Musa AA, Ifenne DI, Essien ES, et al. (1997) The effect of improving maternity services in a secondary facility, Zaria, Nigeria. International Journal of Gynecology and Obstetrics 59: S99-S106.

54. Kayongo M, Butera J, Mboninyibuka D, Nyiransabimana B, Ntezimana A, et al. (2006) Improving availability of EmOC services in Rwanda - CARE's experiences and lessons learned at Kabgayi Referral Hospital. International Journal of Gynecology and Obstetrics 92: 291-298.

55. Kayongo M, Esquiche E, Luna MR, Frias G, Vega-Centeno L, et al. (2006) Strengthening emergency obstetric care in Ayacucho, Peru. International Journal of Gynaecology & Obstetrics 92: 299-307.

56. Kayongo M, Rubardt M, Butera J, Abdullah M, Mboninyibuka D, et al. (2006) Making EmOC a reality - CARE's experiences in areas of high maternal mortality in Africa. International Journal of Gynecology and Obstetrics 92: 308-319.

57. Otchere SA, Binh HT (2007) Strengthening emergency obstetric care in Thanh Hoa and Quang Tri provinces in Vietnam. International Journal of Gynaecology & Obstetrics 99: 165-172.

58. Otchere SA, Kayo A (2007) The challenges of improving emergency obstetric care in two rural districts in Mali. International Journal of Gynaecology & Obstetrics 99: 173-182.

59. Rana TG, Chataut BD, Shakya G, Nanda G, Pratt A, et al. (2007) Strengthening emergency obstetric care in Nepal: The Women's Right to Life and Health Project (WRLHP). International Journal of Gynaecology & Obstetrics 98: 271-277.

60. Santos C, Diante D, Jr., Baptista A, Matediane E, Bique C, et al. (2006) Improving emergency obstetric care in Mozambique: the story of Sofala. International Journal of Gynaecology & Obstetrics 94: 190-201.

61. Mekbib T, Kassaye E, Getachew A, Tadesse T, Debebe A (2003) The FIGO Save the Mothers Initiative: the Ethiopia-Sweden collaboration. International Journal of Gynaecology & Obstetrics 81: 93-102.

62. McQuestion MJ, Velasquez A (2006) Evaluating program effects on institutional delivery in Peru. Health Policy 77: 221-232.

63. Barua A, Waghmare R, Venkiteswaran S (2003) Implementing reproductive and child health services in rural Maharashtra, India: a pragmatic approach. Reproductive Health Matters 11: 140-149.

64. Fort MP, Grembowski DE, Verdugo JC, Morales LC, Arriaga CA, et al. (2011) Implementation and progress of an inclusive primary health care model in Guatemala: coverage, quality, and utilization. Revista Panamericana de Salud Pública 30: 217-224.

65. Marin GH, Silberman M, Uriarte A, Sarijulis M, Ozaeta B, et al. (2011) Healthcare model based on personalised attention: impact on maternal mortality and health system quality. Quality in Primary Care 19: 311-316.

66. Jafari F, Eftekhar H, Fotouhi A, Mohammad K, Hantoushzadeh S (2010) Comparison of maternal and neonatal outcomes of group versus individual prenatal care: a new experience in Iran. Health Care for Women International 31: 571-584.

67. Jafari FE, H.; Mohammad, K.; Fotouhi, A.; (2010) Does Group Prenatal Care Affect Satisfaction and Prenatal Care Utilization in Iranian Pregnant Women? Iranian Journal of Public Health 39: 52-62.

68. Berendes S, Heywood P, Oliver S, Garner P (2011) Quality of Private and Public Ambulatory Health Care in Low and Middle Income Countries: Systematic Review of Comparative Studies. PLoS Med 8: e1000433.

69. Forsberg BC, Montagu D, Sundewall J (2011) Moving towards in-depth knowledge on the private health sector in low- and middle-income countries. Health Policy and Planning 26: i1-i3.

70. Guliani H, Sepehri A, Serieux J (2012) What impact does contact with the prenatal care system have on women's use of facility delivery? Evidence from low-income countries. Social Science and Medicine 74: 1882-1890.

71. Lagarde M, Palmer N (2011) The impact of user fees on access to health services in low- and middle-income countries. Cochrane database of systematic reviews (Online) 4.

72. McNamee P, Ternent L, Hussein J (2009) Barriers in accessing maternal healthcare: Evidence from low-and middle-income countries. Expert Review of Pharmacoeconomics and Outcomes Research 9: 41-48.
